# Detecting Small Size and Minimal Thermal Signature Targets in Infrared Imagery Using Biologically Inspired Vision

**DOI:** 10.3390/s21051812

**Published:** 2021-03-05

**Authors:** Muhammad Uzair, Russell S. A. Brinkworth, Anthony Finn

**Affiliations:** 1 Defence and Systems Institute, UniSA STEM, University of South Australia, Mawson Lakes, SA 5095, Australia; Anthony.Finn@unisa.edu.au; 2 College of Science and Engineering, Flinders University, Tonsley, SA 5042, Australia; russell.brinkworth@flinders.edu.au

**Keywords:** small moving target detection, infrared image processing, biologically inspired vision, unmanned aerial vehicles

## Abstract

Thermal infrared imaging provides an effective sensing modality for detecting small moving objects at long range. Typical challenges that limit the efficiency and robustness of the detection performance include sensor noise, minimal target contrast and cluttered backgrounds. These issues become more challenging when the targets are of small physical size and present minimal thermal signatures. In this paper, we experimentally show that a four-stage biologically inspired vision (BIV) model of the flying insect visual system have an excellent ability to overcome these challenges simultaneously. The early two stages of the model suppress spatio-temporal clutter and enhance spatial target contrast while compressing the signal in a computationally manageable bandwidth. The later two stages provide target motion enhancement and sub-pixel motion detection capabilities. To show the superiority of the BIV target detector over existing traditional detection methods, we perform extensive experiments and performance comparisons using high bit-depth, real-world infrared image sequences of small size and minimal thermal signature targets at long ranges. Our results show that the BIV target detector significantly outperformed 10 conventional spatial-only and spatiotemporal methods for infrared small target detection. The BIV target detector resulted in over 25 dB improvement in the median signal-to-clutter-ratio over the raw input and achieved 43% better detection rate than the best performing existing method.

## 1. Introduction

Detecting small moving targets at long range is a crucial task in many defense and security related applications. Small size unmanned aerial vehicles (UAVs) represent a novel class of small moving targets in our skies. There has been considerable growth in the applications of small UAVs in different areas recently. However, unauthorized use of small UAVs around sensitive facilities may pose safety and security concerns [1,2]. Therefore, seeking new techniques to detect, track and monitor small UAVs at large distances has recently gained considerable research attention.

Among various sensing modalities, thermal infrared imagery is a popular passive modality for small target detection at long ranges. Infrared camera based devices are easily concealable, simpler to operate, and not easy to jam compared to radar based sensors. In contrast to visible spectrum imagers, infrared cameras can work under diverse environmental and weather conditions such as smoke, bad light, snow and dust. Thermal infrared cameras typically produce two dimensional grayscale images by sensing the mid/long (3–14 μm) wavelength electromagnetic spectrum. The captured images represent the thermal profile of the scene being imaged where warmer objects against cooler surroundings provide high contrast regions of interest. Such high contrast characteristic image features have been used for target detection by most state-of-the-art infrared small target detection approaches [3,4,5,6,7,8].

**Figure 1:**
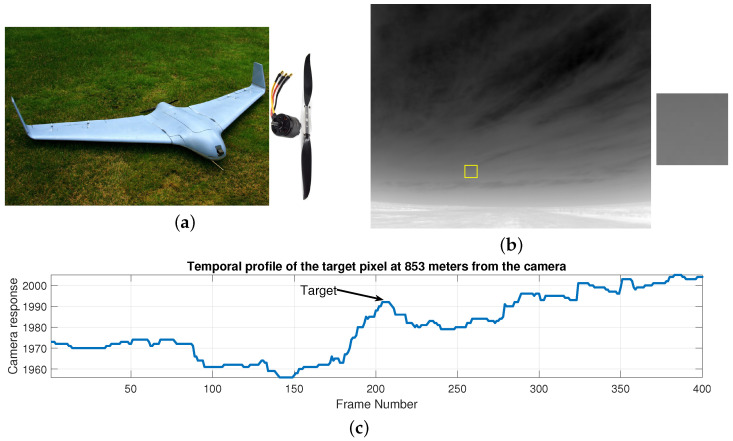
(**a**) A small size Skywalker X8 UAV (79 cm physical length, 212 cm wing span). The main structure is composed of foam and plastic components. The propeller is a 900 Watts small (42 mm CAN diameter) electric brush-less motor. The X8 is a typical example of a small size and minimal thermal signature target. (**b**) Infrared image (minmax scaled) of the target captured using a thermal IR camera (ICI-8640) having a spatial resolution of 640 × 512. The target is at 853 m distance from the camera. Zoomed target region is shown on the right. Due to the small thermal signature of the X8, its brightness and raw spatial contrast are very low compared to the background clutter. (**c**) Raw camera response of the target pixel over time (400 frames). The temporal contrast of the target is also low due to its large distance from the camera.

Detecting physically small targets at long range is challenging due to their unique characteristics such as small physical dimensions, and low thermal and visible signatures. An example of a small size and minimal thermal signature UAV is the Skywalker X8 drone shown in Figure 1a. The main body of X8 does not dissipate any thermal energy on its own but may only reflect some of the incident energy. However, this energy is minimal due to the small physical dimensions of the UAV. Therefore, most of the thermal energy dissipation is due to the small electric motor. Such low thermal energy produces a minuscule thermal signature in the infrared imagery and the resulting target resolution and signal-to-noise-ratio (SNR) is very weak (Figure 1b,c). The SNR degrades drastically when the distance between such a target and sensor increases. Due to design limitations, thermal infrared cameras are typically developed to function at low temporal and spatial resolutions. As a result, fine target texture features are unavailable. Other factors such as sensor noise further reduces the SNR strength. Such low contrast specifications makes accurate detection very challenging for contrast based detection schemes. Additionally, typical real-world scenes also contain *clutter* which are high intensity regions generated by reflected radiations or thermal emissions from background objects. Clutter suppression becomes a necessary task to increase the strength of the signal-to-clutter-ratio. These challenges make detection of small size targets at long range a particularly difficult task, and resolving them simultaneously and efficiently is crucial for high detection performance.

Existing conventional detection approaches solve the problem of infrared small target detection either using only the spatial information available in individual single frames (spatial-only) or using both the spatial and temporal information in the infrared image sequences (spatiotemporal). These methods provide good target enhancement and clutter suppression capability, but assume that the targets are of sufficiently high thermal signature compared to the background elements. Spatial-only detection methods assume that sufficiently high contrast target features are available to be exploited for target modeling. Hence these methods are mostly suitable for relatively higher thermal signature targets. Most of the single frame spatial-only methods apply hand designed spatial filters on the input images to enhance the target region. These filters are commonly computed from the statistics of local pixels. For example, simple techniques such as morphological transformation, max-median and max-mean [9,10] are essentially spatial high-pass filters on local regions to enhance high contrast regions. These methods are computationally lightweight but produce more false-alarms as they sensitive to noise. The more recent local saliency and contrast based approaches [3,5,8,11,12,13,14,15,16] are designed to mimic the attention mechanism of the human visual system. These methods enhance the contrast of a local region with respect to its surround at various local and global spatial scales. However, these approaches also enhance high intensity background clutter elements. The recent matrix factorization based methods [4,17] are more accurate but computationally very expensive. Applying spatial-only approaches to detect long range, small size and minimal thermal signature targets may require a pre-processing step to strengthen the thermal signature of the targets [18,19].

Spatiotemporal detection methods use both the spatial and temporal information available in image sequences to build efficient target detection models. Temporal information is used to highlight moving regions while spatial information is exploited to enhance the highlighted regions. For example, the recursive temporal variance filter [20] and temporal adaptive filtering [21,22] use a combination of temporal band-pass filters to generate a model of the time varying background. Moving regions are then detected by comparing and subtracting the input frames from the model. The detected moving regions are further enhanced by using a spatial contrast operator in conjunction. Other spatiotemporal approaches including the temporal contrast filter [23,24] accomplish the same function with simpler filters and fever computations. Due to the availability of information in both spatial and temporal domains, spatiotemporal methods are more robust compared to spatial-only methods. Despite their efficiency, existing spatiotemporal methods may not show robust adaptation to long-term scene variations due to their model’s susceptibility to error accumulation (Figure 5).

Existing spatial-only and spatiotemporal methods of infrared target detection were mostly developed and tested using synthetic or real datasets of large size targets, e.g., helicopter, ships, aircraft or missiles [3,4,5,8,25,26]. Due to their huge size and high heat dissipation, the level of infrared energy radiated by these objects is significantly greater than small UAVs (such as an X8 drone). For example, an aircraft radiates large amounts of infrared energy produced by components such as the heated engines and metal body parts, and the reflected energy of the sun and sky [27]. When imaged using an infrared camera from a long distance, even though such objects may occupy small number of pixels in the image, their local contrast is sufficiently bright against the background. Hence, traditional target detection methods are readily applicable in such scenarios. In contrast, very little thermal energy is radiated by the small size UAVs that are studied in this paper. As a consequence, their raw brightness (and therefore contrast) is also very low, in addition to occupying very few pixels within the image (Figure 1b). Therefore, with the increase in the target distance from the camera, detection becomes more challenging in this scenario.

Research studies have shown that the biological vision system of small flying insects are exceptionally proficient at noise reduction, contrast enhancement, signal compression and clutter suppression. A multi-stage computation model of the fly vision system was proposed in multiple studies and was shown to possess excellent target enhancement and detection capability in simulated visual environments [28,29]. In this paper, our main goal is to experimentally demonstrate that a four-stage bioinspired vision based target detector [28,29,30] is able to robustly deal with the challenges associated with long range, small size and minimal thermal signature targets in real infrared imagery. To show the superiority of the BIV target detector over existing traditional detection methods, we perform extensive experiments and performance comparisons using high bit-depth real infrared image sequences of small size and minimal thermal signature targets at long ranges. Our experimental data consist of high bit-depth thermal infrared image sequences of real-world scenes. The targets are physically small and minimal thermal singature UAVs (e.g., Skywalker X8) flying against non-stationary cluttered background at long distances from a stationary camera. Our results show that the BIV target detector has superior detection performance compared to 10 conventional spatial-only and spatiotemporal methods.

Our main contributions include:

(1) We perform extensive evaluation and compare the robustness of the BIV target detector with 10 conventional state-of-the-art target detectors using our newly collected infrared imagery. To the best of our knowledge, the BIV target detector analysis for the detection of small size and minimal thermal signature targets is new.
(2) The BIV target detector was previously tested mostly using simulated visible spectrum imagery. As the infrared imagery is generated by a process having a fundamentally different distribution (heat distribution) than the visible spectrum imagery (light distribution), the signal and noise characteristics of the two are significantly different. Our results also confirm that the BIV target detector is just as applicable to inputs from different regions of the electromagnetic spectrum with different data distributions.
(3) Our results show that the spatial-only methods are less reliable for detecting long range, small size and minimal thermal signature targets and that temporal information is beneficial for solving such a problem robustly.
(4) The BIV target detector utilizes highly nonlinear processing stages. Our processing times investigation show that these stages do not come at an increased cost of processing efficiency when compared to the previous existing methods.

## 2. Bio-Inspired Vision Based Target Detector

**Figure 2:**
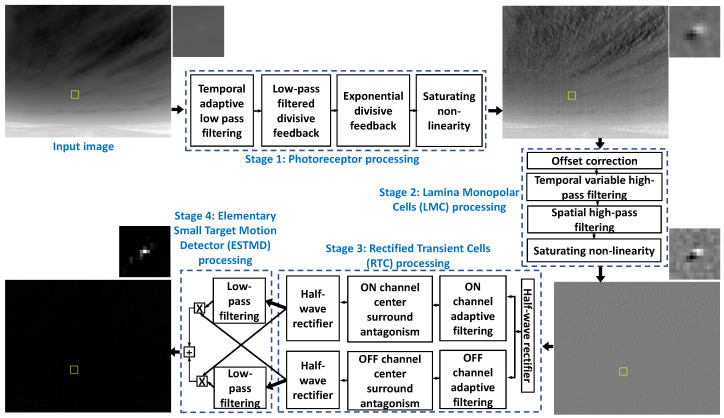
Processing mechanisms and spatial input/output of different stages of the bio-inspired vision based (BIV) target detector. The small size and minimal thermal signature target (X8 drone) is at 520 meters from the camera in this example. The first two stages of the model pre-process the frame to significantly increase the target contrast both spatially and temporally with respect to the background. Stage 2 (LMC) is specifically effective in suppressing the clutter produced by the slow moving clouds and static ground structures. The ESTMD stage outputs a detection map where high values corresponds to detected target regions.

Neurophysiological research have shown that the brain of small flying insects has distinct processing stages for the enhancement and detection of small moving targets [31,32]. One such biologically inspired vision model of small moving target detection consists of four stages for processing a high dynamic range input signal. Each stage is based on a computational model built using the neuronal responses in the target detection pathway of flying insect visual system. The four stages correspond to the operation carried out in the biological photoreceptor cells (PRC), lamina monopolar cells (LMC), rectified transient cells (RTC), and elementary small target motion detector (ESTMD) neurons of the insect visual system. This model has been shown to possess excellent target detection capability in simulated high dynamic range imagery [28] as well in low-dynamic range natural conditions [33]. Figure 2 shows the processing mechanisms involved in each stage of the BIV target detector. The input and output of different stages are also shown for a real-world infrared image of a small size minimal thermal signature target. A brief overview of each stage is provided below. For complete description and implementation details of each stage, we refer the readers to [28,29,32,33,34].

The early two stages of the BIV target detector provide an excellent capability of improving the SNR, suppressing temporal noise and spatial clutter and compressing the signal bandwidth. Pioneering theoretical research on the response of blowfly’s early stage vision was carried out by van Hateren et al. [35,36]. These finding were later incorporated with real physiological measurements from the living neurons of flying insects into the development of a full computational model [28,29,32]. The functions of the real biological cells were modeled digitally with cascaded adaptive filters to simulate the signal pre-processing in the insect early vision [28,29].

More specifically, stage 1 of the BIV target detector is a numerical model of the processes in the photoreceptor cells of small flying insects for temporal signal enhancement. This stage performs adaptive temporal processing of the input visual data on a pixel-wise basis. Stage 1 suppresses temporal noise, enhances the temporal signature of the target and adaptively adjusts scene luminance in low and high intensity regions. The output of stage 1 has a reduced redundancy compared to the input visual data which is useful to accurately encode the information in the limited neuronal bandwidth. In other words, stage 1 improves the moving target detectability without losing potentially useful motion features [37].

The second order LMC neurons in the brain of small flying insects provide the basis for stage 2 of the BIV target detector [28,29]. The LMC processing applies spatiotemporal, SNR adaptive high-pass filtering on the photoreceptor outputs [38,39]. The LMC enhances spatial target contrast while significantly suppressing the spatiotemporal clutter. The LMC also removes spatiotemporal redundancy which reduces the response from the redundant scene regions such as high intensity stationary background structures.

Stage 3 models the rectified transient cells (RTC) of the fly brain. RTC responses have been shown to be well-suited for small target motion enhancement in the target detection pipeline of insect visual system [32]. The main function of these cells includes full-wave rectification to produce transient ON/OFF channels and adaptive temporal filtering of the resultant channels of the incoming signals from the LMC. The ON/OFF streams are processed temporally using a fast adaptive filtering mechanism. Specifically, a nonlinear filter is used to determine an adaptation state which approximates the cellular “fast depolarization and slow repolarization” mechanism to subtractively inhibit the unchanged pass-through signals. Such non-linear temporal antagonism suppresses fluctuating textural features while enhancing the actual moving features. Spatial antagonism has also been incorporated to further enhance the moving target features in spatial domain [28].

Stage 4 is a model of the elementary small target motion detectors (ESTMD). The processing involved in this stage follows a theoretical model of the input to the Small Target Motion Detector (STMD) [40] neuron and is not based on the actual neurophysiological recordings from within the brain of an insect. ESTMDs implements the elementary motion detector [41] using the two processed channels from the RTC. The channels are combined by delaying one channel via a first-order low-pass filter and multiplying with the un-delayed opposite channel. The sensitivity to both light and dark targets can be combined by the delay and recombination of the relevant contrast polarity. This type of model is size and velocity tuned which means it is more suitable for slow moving small targets. As the targets are bright in real-world infrared imagery, we adapted the stage 4 of the BIV target detector by integrating the responses of the dark and bright detection channels. The final output of the BIV target detector is a real-valued detection map which is segmented using a fixed or adaptive threshold to find target pixels.

## 3. Performance Evaluation

### 3.1. Infrared Image Data

For performance evaluation we collected a real-world scenes database containing small size and minimal thermal signature targets. An ICI 8640 thermal IR camera was used for image capture. The camera operated in the spectral range of 7–14 μm and produced 640 × 512, 14-bit images at 30 frames per seconds. A small size, lightweight and minimal thermal signature UAV (Skywalker X8 Figure 1) flying at a speed of 65–70 km/h across the stationary camera’s FOV (50° × 37.5°). The distance of the UAV from the camera was recorded by a small global positioning system (GPS) device. Typical scene clutter elements included ground elements responses, moving clouds signatures and sky based reflections.

**Figure 3:**
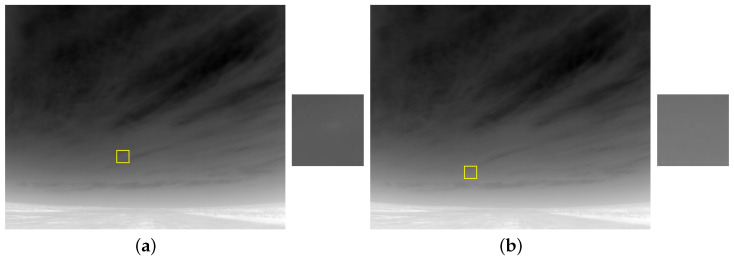
Infrared images of small size and minimal thermal signatures target at long range ((**a**) 520 m, (**b**) 853 m). Zoomed target regions are shown on the right. The target’s spatial contrast is minimal and the scene contains cluttered bright background regions. (For display, images are minmax normalized).

Figure 3a,b shows two sample images from our database. The target was at 520 m (Figure 3a) and 853 m (Figure 3b) from the sensor. Various detection difficulties are present in these example frames. For instance, because of its minimal physical size and minimal thermal signature, the target brightness is very low. This problem is more pronounced at the higher distance frame. Additionally, the thermic radiation from the cluttered regions is high (Figure 3b), which makes it very hard to discriminate the target from the clutter. 

For experiments reported in this paper, 681 infrared images were processed. The spatial resolution of the target in these images roughly ranges from a pixel up to around 5 × 5 pixels. We split the dataset into three subsets of short, medium and long ranges. The target sensor distance was 500–700 m, 700–800 m and 800–900 m in the short, medium and long range sets, respectively. There number of images were 400, 131 and 150 in the short, medium and long range sets, respectively. The medium range set was used as a development set for parameter tuning purposes only. The ground truth was manually established with the help of the GPS data and a MATLAB tool.

### 3.2. Existing Conventional Methods for Comparison

We compared the performance of the the BIV target detector with 10 conventional state-of-the-art spatial-only and spatiotemporal methods of detecting targets in infrared imagery.

#### 3.2.1. Spatiotemporal Methods

Five existing spatiotemporal methods were employed in our study. These included the recursive temporal variance filter (RVF) [20], the temporal contrast filter (TCF) [24], the local spatiotemporal contrast (STLC) [42], the multi-scale patch contrast temporal variance (MPCMTVF) [43] and the center surround total differential index (CSTDI) method [19]. In the spatiotemporal category, the baseline method TCF, RVF and STLC have shown robust target detectability performance in infrared data. CSTDI and MPCMTVF are the latest methods.

The spatiotemporal methods TCF, RVF, STLC and CSTDI apply a two stage detection pipeline. In the first stage moving scene regions are detected using a temporal filter. In the second stage, a spatial filter is applied to reject clutter and localize targets. Specifically, the RVF method first uses a temporal variance filter estimated from the per-pixel running variance and then localizes the targets using a max-median spatial filter. The TCF method models the background by a min filter estimated from a local history of frames. The moving regions are detected by subtracting the input frame from the background model. To localize the targets, a spatial filter that maximizes the local signal-to-clutter-ratio is applied. Instead of using the min filter, the STLC method models the background using the mean filter and in conjunction applies a special filter based on the absolute difference between a center pixel and its local neighborhood. The CSTDI method uses a bio-inspired temporal pre-processing (first two stages of the BIV target detector) to enhance the temporal signature of the targets and then applies a center-surround differential spatial filter for target localization. The MPCMTVF method applies a spatial contrast enhancement operator MPCM (multiscale patch based contrast measure) in conjunction with the RVF filter.

#### 3.2.2. Spatial-Only Methods

Five conventional spatial-only methods for the detection of small targets in infrared imagery were employed. These included in our study were the multiscale top-hat morphological transform (MTH), the multiscale local contrast method (MLCM) [3], the average of absolute gray difference method (AAGD) [44], the infrared patch-image based matrix factorization method (IPI) [4] and the recent facet kernel and Random Walker [5] approach. Overall, we tested both simple baseline approaches as well as the more recent state-of-the-art Random Walker [5] approach.

The multiscale top-hat morphological transform (MTH) uses several scales of a morphological structuring element to enhance small size high intensity features through top-hat morphological filtering. The multiscale local contrast method (MLCM) [3] is a simple and effective method for computing the local contrast. MLCM uses dense sliding windows that quantify the gray level contrast of each pixel with respect to its surroundings using gray value ratios. The average of absolute gray difference method (AAGD) [44] also employs sliding window based technique to compute the contrast of each pixel but uses gray value differences instead of ratios and two levels of inner and outer windows. The infrared patch-image [4] method divides the image into dense overlapping patches to construct a data matrix. A sparse target image matrix and a low rank background image matrix are then recovered from the data matrix via a mixed norm optimization problem. The accelerated proximal gradient (APG) algorithm was used to find the solution. The Random Walker [5] method first applies local statistics based filtering for removing pixel sized noise. Next, the image is filtered using a facet kernel that enhances the contrast of the bright target features. Finally, a Random Walker [45] based segmentation algorithm is employed to segment the target and background regions.

### 3.3. Performance Assessment Metrics

To quantify the target contrast enhancement and background clutter suppression ability of different methods, we adopt a commonly used quantitative measure referred to as the signal-to-clutter-ratio (SCR) gain [46] as defined below:

(1) $$\begin{array}{c} \mathrm{SCR}=\frac{|\mu_{t}-\mu_{b}|}{\sigma_{b}},\;\;{\mathrm{SCR}}_{gain}=20\log_{10}\frac{{\mathrm{SCR}}_{output}}{{\mathrm{SCR}}_{input}}\;\mathrm{dB}, \end{array}$$

where $\mu_{t}$, $\mu_{b}$ and $\sigma_{b}$ represent the average target luminance, average of the background luminance and standard deviation of the background, respectively. Similarly, ${\mathrm{SCR}}_{input}$ represents the input image SCR while ${\mathrm{SCR}}_{output}$ denotes the detection map’s SCR. These quantities are usually computed from a target window and a background window around the target. We imposed a tight constraint on the extent of these windows by using a 5 × 5 window around the target and using the rest of the image as background window. Such a constraint allows us to measure a global frame level SCR gain for each method.

The target detection performance is quantified by the commonly used receiver operating characteristic (ROC) curve generated from the detection rate (DR) and false alarm rate (FAR) [47] quantities calculated as:

(2) $$\begin{array}{c} \mathrm{DR}=\frac{T_{p}}{T_{a}},\\ \mathrm{FAR}=\frac{F_{p}}{N}. \end{array}$$

where $T_{p}$ defines the true detections, $T_{a}$ defines the actual total targets, $F_{p}$ defines the number of false positive detections (incorrectly segmented pixels) and *N* denotes the number of total pixels in the input image. Detections within a radius of five pixels of the ground truth’s center were considered correct. The ROC was generated by segmenting the detection maps of each method by varying a threshold sensitivity parameter α in small steps and measuring the above quantities for each step. The bottom left conservative part of such an ROC plot is the typical desired operating region (lower false alarm rate) of most object detection systems [48]. This means that a method is favored if it can provide a higher detection rate at a minimum false alarm rate.

### 3.4. Experimental Setup

The Matlab implementations of Random Walker, IPI and CSTDI methods were provided by the respective authors. For the MTH, AAGD, MLCM, TCF, RVF, MPCMTVF and STLC methods we used our own implementations based on the respective papers. Manually parameter tuning was done for all methods using the medium range set. A common approach for parameter search is to optimize one or more performance metrics. We adopted the detection rate at a false alarm rate of $10^{-5}$ as our optimization metric. Compared to other conventional approaches, the performance of the BIV target detector is less sensitive to parameter tuning. This is because the BIV model is adaptive in nature and gradually adapts its parameters over time to the characteristics of the input data. This selfadaptation is a key feature of the biological systems [29,37,49]. Such inherent adaptation enables the BIV target detector to operate consistently over a wide variety of scenes [50] with increased robustness to noise [51].

Table 1 summarizes the parameter settings of different methods. The parameters of the spatiotemporal methods were chosen as follows. The history frame buffer size was set to 30 for the TCV, RVF and STLC methods. The base segmentation parameter of the TCF was calculated as 40% of the peak response in the filtered output and 8 NN clustering was used for target segmentation filter. The spatial window extent in the STLC was 5. MPCMTVF used the same temporal filter as the RVF. The scales of the STDI were set to 8, 16 and 24 and median scale fusion was used.

The parameters of the spatial-only methods were chosen as follows. A disk-structuring element was used in the MTH and the scales were set to {3,5,7,15,31,61}. The MLCM window scales were 5,7,9 and 11. The outer AAGD window scales were {9,13,17} while the inner AAGD window scales were {1,3,5}. In the IPI algorithm, the patch size was 80×80 while the step-size was 14. The APG minimizer was executed with default parameters as in [4]. In the Random Walker algorithm, the top level segmentation threshold was 4.

### 3.5. Target Enhancement Comparison (Temporal)

Figure 2 shows the visual spatial outputs of different stages of the BIV model. The target is at 520 m from the camera. The photoreceptor stage generates a bi-phasic signature of the moving target with a bright leading and a dark trailing edge. It also provides scene brightness normalization across different regions. The photoreceptor generated signature is further sharpened by the spatial center surround antagonism based mechanism of the LMC while the variable spatiotemporal high pass filters in the LMC stage suppresses the response of the clutter regions. Thus, the first two stages of the model provide an excellent pre-processing of the input data. The bi-phasic output of the LMC is processed by the RTC stage via ON and OFF channel processing. A final detection map is generated by the ESTMD stage by correlation based aggregation of the On and OFF RTC channel outputs.

**Figure 4:**
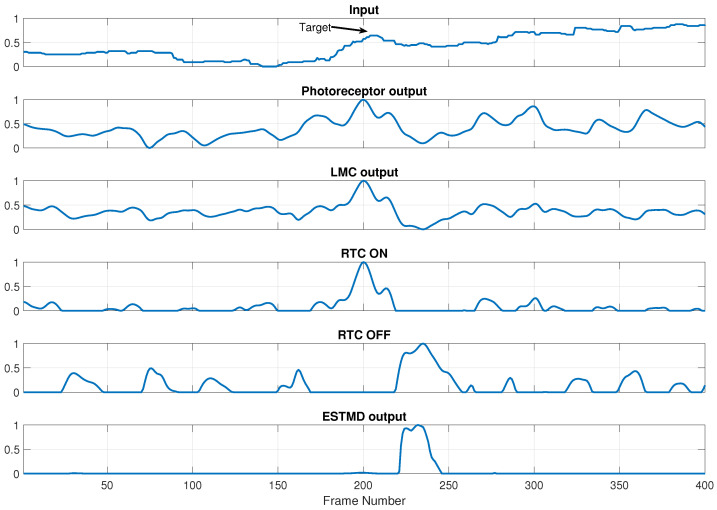
Temporal profiles of the normalized responses of the BIV stages to the input target pixel shown in Figure 1c. The multistage processing of the BIV has significantly increased the temporal contrast of the target.

Figure 4 shows the temporal response of each BIV stage to the input signal of the target at 853 m (Figure 1c). Each subsenquent stage of the BIV has increased the target’s weak temporal contrast and suppressed unwanted temporal clutter. The final stage output is an enhanced target signature which can be segmented from the background with high accuracy via simple threshholding.

**Figure 5:**
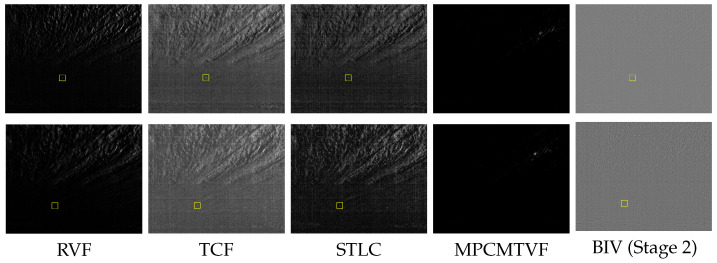
Temporal filtering results of the spatiotemporal methods for the images in Figure 3a (top row) and Figure 3b (bottom row). Conventional approaches failed to cope with the slow cloud motion. The MPCMTVF could not detect the target in both examples. In comparison, the first two stages of the BIV target detector (LMC output) maximized both the SNR and SCR of the target significantly better than the other methods. CSTDI is not shown separately as it uses the LMC output in its temporal filtering stage.

A qualitative comparison of the moving target enhancement and clutter suppression strength of the spatiotemporal detection methods is shown in Figure 5. For a fair comparison, the outputs of the temporal filtering (motion enhancement stage) of each spatiotemporal method is shown. Although, the existing traditional methods enhanced the target motion, they failed to robustly model the slow moving clutter generated by the clouds. This is because their simpler temporal filtering strategies accumulate errors over the long-term. This leads to more false positive generation by the subsequent target segmentation stages. On the other hand, by incorporating biologically based variable and adaptive temporal filtering mechanisms, the BIV model has completely suppressed both the moving and static clutter regions while enhancing the target. Such simultaneous clutter suppression and target enhancement is the key to achieving correct detections and lower false alarms by the subsequent detection stages. Although the TCF, RVF and STLC has provided target enhancement when the target is nearer to the camera (520 m), they still generated many false positives in this range; and the existing algorithms suffers greatly at long ranges (853 m) by failing to enhance the target and instead enhancing the clutter. The MPCMTVF completely failed at both distances. This is because MPCMTVF suppressed the low SNR target in its early stage spatial filtering prior to the temporal motion modeling. These results suggest the superiority of the BIV model over existing methods for clutter suppression and target enhancement.

### 3.6. Target Enhancement Comparison (Spatiotemporal)

**Figure 6:**
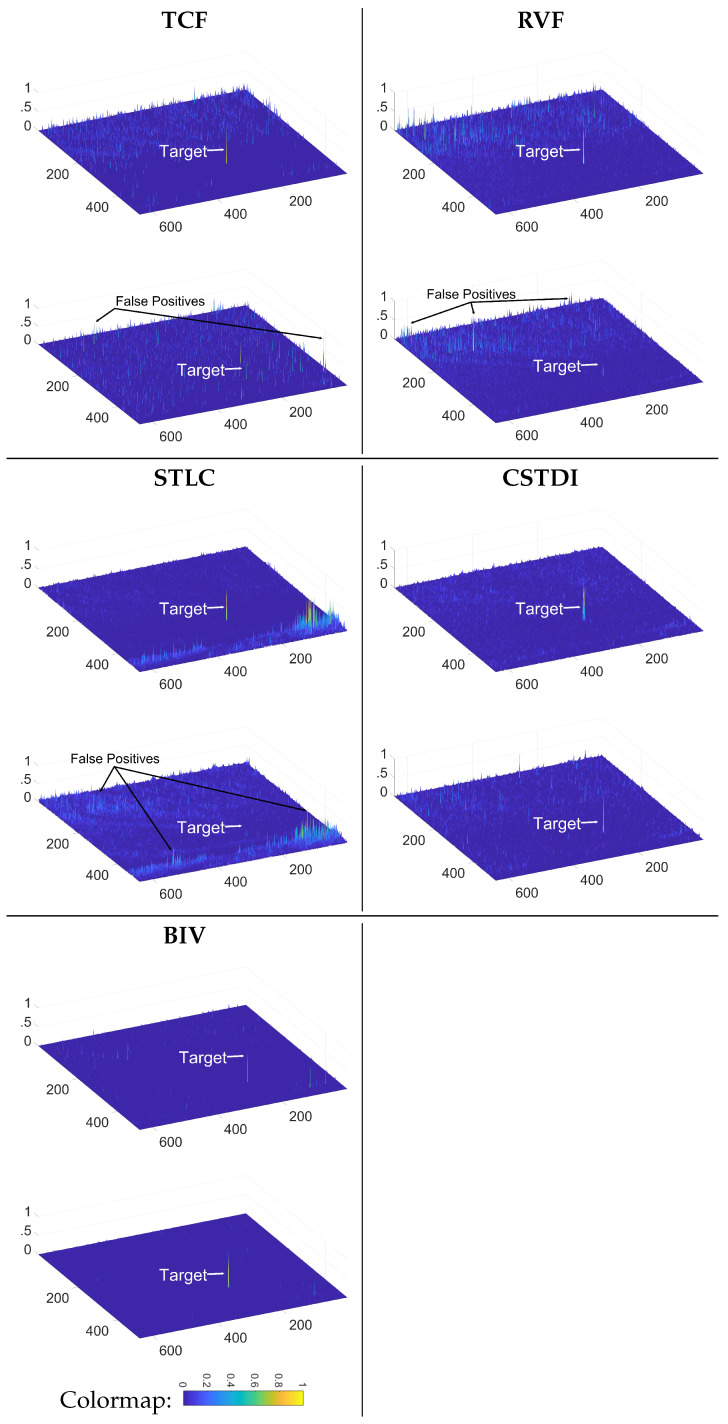
Detection outputs of spatiotemporal methods. The upper detection map in each cell is for the image in Figure 3a while the lower detection map is for the image in Figure 3b. Traditional approaches produced greater false alarms and could miss the target. The biologically inspired methods (CSTDI and BIV) overcome both issues showing better robustness than the other methods, with the BIV target detector even better at supressing false alarms than the CSTDI.

Figure 6 shows the outputs of different spatiotemporal approaches applied on the images in Figure 3a,b. The output map of MPCMTVF is not displayed as it was not able to detect the target in any of the two frames. Peaks in the output maps indicate possible target detections. For the short range target, although existing spatiotemporal methods produced a high target response they generated many false peaks for the cluttered regions. For the long range target, the responses generated by the existing methods are very weak. The STCL method could not produce any target response at longer range. In contrast, the BIV target detector generated correct high peak responses for the targets while at the same time very low false peaks at both short and long ranges. The CSTDI takes advantage of the first two stages of the BIV for spatiotemporal pre-processing. Therefore, CSTDI produces more accurate detection maps compared to previous traditional methods. However, the BIV target detector is more robust due to two more spatiotemporal stages (RTC, ESTMD) after the LMC which can suppress spatiotemporal noise much better than CSTDI. Therefore, the BIV produces cleaner detection maps. This makes BIV a better choice for applications requiring a higher level tracking module for accurate UAV track generation.

**Figure 7:**
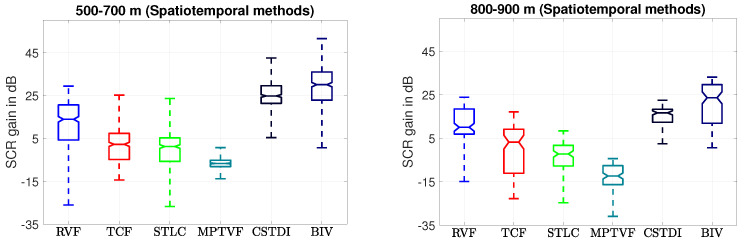
SCR gain distribution of the spatiotemporal detection methods. The BIV target detector provides the highest SCR gain compared to others.

To quantitatively assess the target enhancement and clutter suppression strength of the spatiotemporal methods, we computed the ${\mathrm{SCR}}_{gain}$ for each raw input frame and its corresponding output detection map via Equation (1). The overall distributions of the ${\mathrm{SCR}}_{gain}$ for different ranges are shown in Figure 7 as a notched boxplots [52]. The median ${\mathrm{SCR}}_{gain}$ (middle horizontal box lines) of the BIV target detector is significantly higher than the other methods with no frames having negative ${\mathrm{SCR}}_{gain}$. This means that in no frames did the BIV processing have a negative affect on the detectability of the target compared to the raw input data. The upper and lower box edges defines the first and the third quartiles, respectively. The BIV target detector has higher values than the other methods for these quantities also. The BIV target detector provides 21.3% and 41.7% better median SCR than the CSTDI for the 500–700 m and 800–900 m range, respectively. The box plots also shows that performance gain of the BIV target detector over other methods is statistically significant with a confidence of 95%. This is because there is no overlap between the box notches of the BIV target detector and existing methods [52].

### 3.7. Detection Performance Comparison: Spatiotemporal Methods

**Figure 8:**
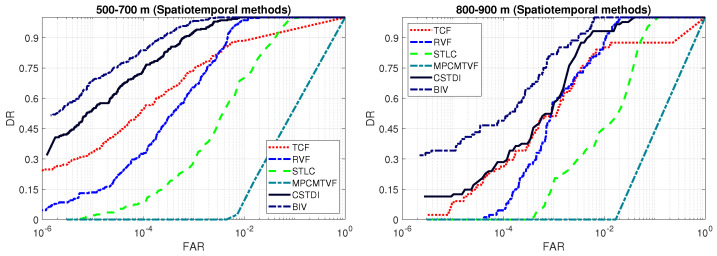
ROC curves comparison: spatiotemporal methods (logarithmic x-axis). Existing methods are outperformed by the BIV target detector on both ranges.

Figure 8 compares the ROC curves of the spatiotemporal methods. The conventional approaches were outperformed by the BIV target detector on both mid range and long range datasets. For example, at a small false alarm rate of $10^{-5}$, the TCF, RVF, MPCMTVF, STLC and CSTDI obtained a detection rate of 0.33, 0.13, 0, 0.02 and 0.54, respectively, in the case of 500–700 m range. The BIV target detector achieved a 27.7% higher detection rate (0.69) over the best competing method (CSTDI). Similarly, the BIV achieved a 209.0% better detection rate of 0.34 on the 800–900 m range over the best competing method CSTDI.

**Figure 9:**
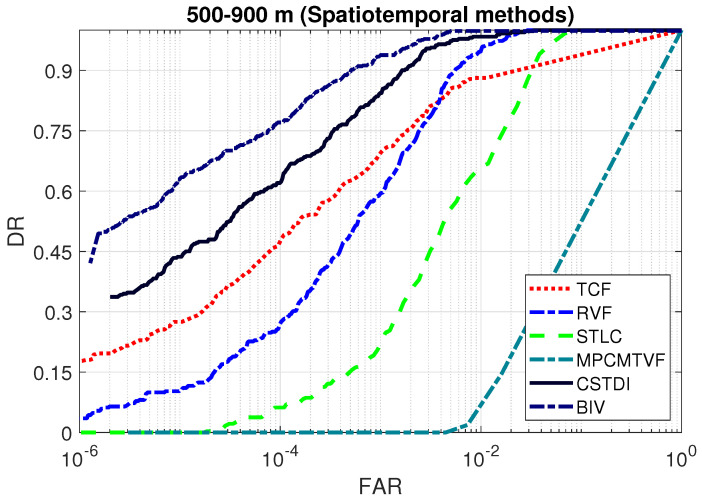
ROC curves comparison: spatiotemporal methods (logarithmic x-axis). Existing methods are outperformed by the BIV target detector on the combined range showing that the improved response of the BIV model is not due to overfitting of parameters for each range.

In order to confirm that the BIV was not specifically tuned to detecting targets under one particular condition, we also evaluate it on the combined short and long ranges (500–900 m). For all methods, the parameters used for the integrated range and the separate ranges were the same. Figure 9 the detection performance comparison on the combined range. The BIV target detector outperform the conventional methods in this experiment also. On the integrated range, at $10^{-5}$ FAR, the TCF, RVF, MPCMTVF, STLC and CSTDI provided a detection rate of 0.27, 0.10, 0, 0 and 0.44, respectively. The BIV attained a detection rate of 0.63 which shows an increase of 43.1% over the next best performing method, CSTDI.

### 3.8. Detection Performance Comparison: Spatial-Only Methods

We perform two experiments to assess the detection performance of the conventional spatial-only methods. In the first experiment, the inputs to these methods are raw image frames. In the second experiment, we pre-process the images with the first two stages of the BIV target detector and then apply existing spatial-only methods for target detection.

**Figure 10:**
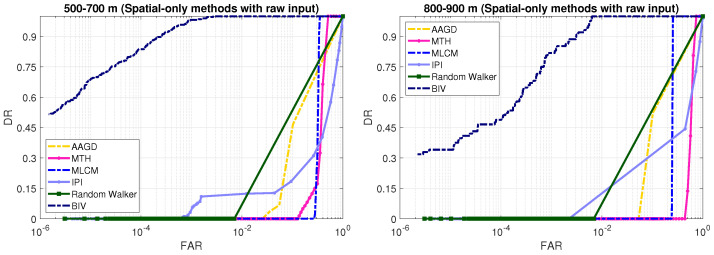
ROC curves comparison: spatial-only approaches (logarithmic x-axis). The spatial-only methods failed at both ranges when the input was raw.

Figure 10 shows the detection results of the five conventional spatial-only methods when their input images were raw. Existing spatial-only methods completely failed on the raw input images. Although, previously these methods have shown good detection performance [3,4,5] on datasets of physically large high-thermal-profile targets, these methods failed to deal with small size and minimal thermal signature targets at long ranges. These results indicate that using only spatial information is not sufficient to solve the problem at hand.

**Figure 11:**
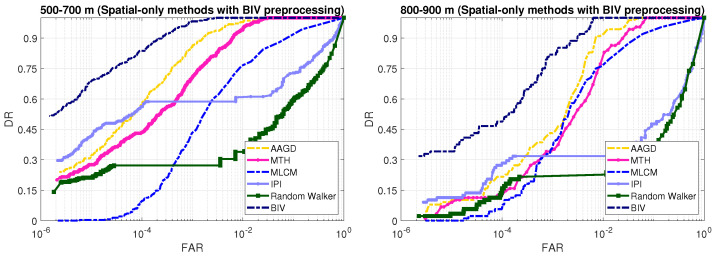
Detection results of the spatial-only approaches where the input images are preprocessed with the first two stages of the BIV target detector. The BIV preprocessing has significant increased the performance of these methods compared to their raw input versions (Figure 10). In spite of the improvement gained by using the BIV pre-processing, the existing methods are still out performed by the BIV target detector at both ranges.

Figure 11 shows the increase in the detection rate performance of the spatial-only methods when their input is pre-processed with the second stage of the BIV (LMC). The spatiotemporal pre-processing of the first two stages of the BIV significantly boosted the detection rates of the conventional spatial-only techniques. However, these methods were still outperformed by the BIV target detector. With the BIV pre-processed frames, the IPI obtained better results than the other spatial-only approaches. However, IPI is computationally expensive. For example, IPI took over 2500× longer to compute than the BIV.

It should be noted that the BIV target detector is not limited only to the problem of detecting small size and minimal thermal signature targets. It is also just as applicable for the detection of large sized and strong-thermal-signature targets at long range. In addition, the pixel-wise enhancements offer up improved contextual features that can be extracted and passed to other algorithms.

### 3.9. Compute Time

The average execution times (seconds) per image were computed using Matlab implementations of the algorithms running on a 3.4 GHz processor (Corei7) and 16 GB RAM computer. Table 2 summarizes the computational time of different methods. The spatiotemporal methods TCF, RVF, MPCMTVF, STLC and CSTDI took 3.7, 3.6, 0.35, 0.15 and 0.13 s per frame, respectively. The spatial-only methods IPI, MLCM, Random Walker, MTH and AAGD took 320, 0.33, 0.27, 0.13 and 0.05 s per frame, respectively. The un-optimzed implementation of the BIV target detector took 0.13 s per frame. The execution times of individual stages (stage one to four) of the BIV target detector were 0.01, 0.003, 0.11 and 0.003 s per frame, respectively. This shows that the BIV target detector is much faster than the other spatiotemporal methods, while achieving significantly better detection performance. This confirms that the superior detection performance of the BIV target detector is not at the expense of increased computational burden.

## 4. Conclusions

The performance of a practical small target detection system relies heavily on solving two problems simultaneously and robustly: maximizing the signal-to-noise-ratio to achieve high detection rates at longer ranges; and maximizing the signal-to-clutter-ratio to reduce the false alarms. These problems become more important when dealing with small size and minimal thermal signature targets such as small UAVs at long range.

This paper presents a biologically inspired vision (BIV) based four-stage computational model that efficiently overcomes these issues. Our experiments with high bit-depth, real-world infrared imagery confirm that the BIV target detector is superior to other traditional methods in terms of target enhancement and clutter suppression capability and achieves significantly better detection rates at low false alarm rates. While previous work has shown that the BIV can be used for target detection in simulated visual scenes [28] and the first two stages of the model improve the detection of small size and low thermal signature infrared targets [19], this is the first time the full target detection processing pipeline of the BIV has been shown to function on real-world infrared imagery.

Our results also conclude that the spatial-only methods perform worse than the spatiotemporal methods for the detection of small size and minimal thermal signature targets at long range. This is partly because the spatial-only methods focus on maximizing the signal-to-clutter-ratio only and hence are unable to simultaneously maximize the SNR of the minimal thermal signature targets at long ranges. In contrast, by exploiting target motion information, the spatiotemporal methods can improve the SNR and extend the detectability range.

The focus of our current study is on target detection using a stationary camera. In order to deal with clutter due to camera motion, additional processing stages will be required for target detection from a non-stationary platform. The BIV model has been extended to deal with camera motion in one such simulation study [30]. Our future work involves extending the BIV target detector to real-world data captured by a moving platform.

## Figures and Tables

**Table 1:** Parameter settings of different methods.

Method	Parameters
MTH	Structuring element scales = {3,5,7,15,31,61}
MLCM	window scales = {5,7,9,11}
AADG	outer window scale = {9,13,17}, inner window scale={1,3,5}
IPI	patch size = 80 × 80, solver = APG
Random Walker	top level segmentation threshold = 4
TCF	history buffer = 30, seed segmentation threshold = 40%, 8NN clustering
RVF	history buffer = 30
STLC	history buffer = 30, spatial window extent = 5
MPCMTVF	history buffer = 30, same filter as in RVF
CSTDI	scales = {8,16,24}, medium scale fusion

**Table 2:** Comparison of the compute time in seconds per frame (640 × 512 resolution).

**Spatial-only methods**						
Method	IPI	MLCM	Random Walker	MTH	AADG	
Compute time (s/frame)	320	0.33	0.27	0.13	0.05	
**Spatiotemporal methods**						
Method	TCF	RVF	MPCMTVF	STLC	CSTDI	BIV
Compute time (s/frame)	3.7	3.6	0.35	0.15	0.13	0.13
